# Identification of *Aedes aegypti* cis-regulatory elements that promote gene expression in olfactory receptor neurons of distantly related dipteran insects

**DOI:** 10.1186/s13071-018-2982-6

**Published:** 2018-07-11

**Authors:** Keshava Mysore, Ping Li, Molly Duman-Scheel

**Affiliations:** 1 Department of Medical and Molecular Genetics, Indiana University School of Medicine, 1234 Notre Dame Avenue, Raclin-Carmichael Hall, South Bend, IN 46617 USA; 2 The University of Notre Dame Eck Institute for Global Health, Notre Dame, IN 46556 USA; 3 Department of Biological Sciences, The University of Notre Dame, Notre Dame, IN 46556 USA

**Keywords:** Mosquito, Sensory, Antenna, Neuron, Enhancer, FAIRE, *Aedes aegypti*, *Drosophila melanogaster*, Dengue, Zika

## Abstract

**Background:**

Sophisticated tools for manipulation of gene expression in select neurons, including neurons that regulate sexually dimorphic behaviors, are increasingly available for analysis of genetic model organisms. However, we lack comparable genetic tools for analysis of non-model organisms, including *Aedes aegypti*, a vector mosquito which displays sexually dimorphic behaviors that contribute to pathogen transmission. Formaldehyde-assisted isolation of regulatory elements followed by sequencing (FAIRE-seq) recently facilitated genome-wide discovery of putative *A. aegypti* cis-regulatory elements (CREs), many of which could be used to manipulate gene expression in mosquito neurons and other tissues. The goal of this investigation was to identify FAIRE DNA elements that promote gene expression in the olfactory system, a tissue of vector importance.

**Results:**

Eight *A. aegypti* CREs that promote gene expression in antennal olfactory receptor neurons (ORNs) were identified in a *Drosophila melanogaster* transgenic reporter screen. Four CREs identified in the screen were cloned upstream of *GAL4* in a transgenic construct that is compatible with transformation of a variety of insect species. These constructs, which contained FAIRE DNA elements associated with the *A. aegypti odorant coreceptor* (*orco*), *odorant receptor 1* (*Or1*), *odorant receptor 8* (*Or8*) and *fruitless* (*fru*) genes, were used for transformation of *A. aegypti*. Six *A. aegypti* strains, including strains displaying transgene expression in all ORNs, subsets of these neurons, or in a sex-specific fashion, were isolated. The CREs drove transgene expression in *A. aegypti* that corresponded to endogenous gene expression patterns of the *orco*, *Or1*, *Or8* and *fru* genes in the mosquito antenna. CRE activity in *A. aegypti* was found to be comparable to that observed in *D. melanogaster* reporter assays.

**Conclusions:**

These results provide further evidence that FAIRE-seq, which can be paired with *D. melanogaster* reporter screening to test FAIRE DNA element activity in select tissues, is a useful method for identification of mosquito cis-regulatory elements. These findings expand the genetic toolkit available for the study of *Aedes* neurobiology. Moreover, given that the CREs drive comparable olfactory neural expression in both *A. aegypti* and *D. melanogaster*, it is likely that they may function similarly in multiple dipteran insects, including other disease vector mosquito species.

## Background

Mosquito control is the primary mechanism for preventing dengue, yellow fever, chikungunya and Zika, all of which result from viruses transmitted by the daytime-biting mosquito *Aedes aegypti*, which is closely associated with humans and their urban dwellings. The emergence of insecticide resistance and a lack of support for mosquito control programs compromise current strategies for managing mosquitoes [1]. It is therefore critical that we identify new tools to both study mosquito biology and combat these insect vectors of human disease-causing pathogens. Mosquito behaviors such as blood meal acquisition, courtship and oviposition have attracted the attention of biologists for years. Knowledge of the neurogenetic basis for these and other behaviors would facilitate modification of the behaviors for vector and disease control. Unfortunately, analysis of the neurogenetic basis for insect behavior has largely been restricted to *Drosophila melanogaster*, a genetically-tractable dipteran insect and genetic model organism [2]. While hundreds of neuron-specific GAL4 driver lines enable sophisticated perturbation of the fruit fly nervous system [3, 4], we lack comparable genetic tools for analysis of the neurophysiological basis of behaviors in most insects, including mosquitoes.

Likewise, knowledge concerning the extent of sexual dimorphisms in the structure of the central and sensory nervous systems, the regulation of sex-specific behaviors by sexually dimorphic neurons, as well as the developmental genetic basis for sexually dimorphic behaviors is limited in all organisms, even *D. melanogaster*, but especially in non-model organisms, including mosquitoes [2, 5]. Advancements in the methodology for studying mosquito neurogenetics would help to elucidate the genes that regulate mosquito sexual dimorphism, including the development and function of neural circuitries that promote host-seeking, blood meal acquisition and feeding behavior, mating and oviposition, all of which may represent genetic targets for vector control [2]. Studies in *D. melanogaster* have begun to reveal the genetic mechanisms that underlie sexually dimorphic behavior in insects. For example, sex-specific splicing of the *D. melanogaster fruitless* (*fru*) gene, which encodes a transcription factor, results in male- and female-specific splice forms that contribute to sexually dimorphic behaviors such as courtship and aggression in flies [6–9]. Recent studies in the olfactory system have demonstrated that the actual expression of *fru* is also sexually dimorphic, and that *fru* expression serves as a molecular marker for neurons participating in sex-specific behaviors [10]. The detection of sex-specific splice forms of *A. aegypti fru* suggests that it functions as a modulator of sexually dimorphic behavior in *A. aegypti* [11], but the function of this gene has not yet been directly assessed in mosquitoes.

Although the GAL4-UAS binary system for manipulation of gene expression in *D. melanogaster* neurons has been introduced in *A. aegypti* [12], very few GAL4 lines are currently available. This is largely due to the lack of known CREs in mosquitoes. FAIRE-seq, has emerged as a powerful high-throughput tool for global CRE discovery [13]. FAIRE results in the preferential recovery of open chromatin DNA fragments that are not bound by nucleosomes, an evolutionarily conserved indicator of regulatory activity, which are then sequenced through next-generation sequencing [13–16]. We recently utilized FAIRE-seq to profile open chromatin and identify regulatory elements throughout the genome of *A. aegypti* [17]*.* The results of this investigation [17] provided evidence that FAIRE-seq is a powerful tool for identification of regulatory DNA in the mosquito genome. We are therefore mining the FAIRE-seq data set for regulatory elements that function in tissues of vector importance.

Here, we describe the identification and characterization of CREs that drive gene expression in the olfactory system, a sensory system that is critical for many sexually dimorphic mosquito behaviors related to mosquito reproduction and pathogen transmission [2]. The first phase of the study exploits the genetic tractability of *D. melanogaster*, in which transgenic generation is quick, straightforward, and economical. *Drosophila* reporter assays permitted analysis of *A. aegypti* FAIRE DNA elements of interest, leading to identification of CREs that promote gene expression in antennal olfactory receptor neurons (ORNs). Characterization of the *Drosophila* reporter lines facilitated down-selection of four elements for the direct transformation of *A. aegypti.* CREs that promote gene expression in all *A. aegypti* antennal ORNs, subsets of these neurons, as well as in a sex-specific manner, were identified. The results of this study demonstrate that the regulatory elements function comparably in two distantly related insects, suggesting that they might be used for modification of gene expression, including sex-specific gene expression, in the olfactory systems of *A. aegypti* as well as additional mosquito species and other dipteran insects. These tools, particularly the sex-specific gene driver, may promote the elucidation of new methods for control of disease vector mosquitoes.

## Methods

### Mosquito rearing

Mosquitoes were reared as previously described [18]. A membrane blood-feeding system was employed in conjunction with commercially supplied sheep blood (Hemostat Laboratories, Dixon, CA). Following establishment of each transgenic strain, an eye-specific genetic marker was selected in subsequent generations for continued maintenance of the strain. Egg libraries are also being maintained for the transgenic strains.

### *Drosophila melanogaster* transgenic reporter generation and analysis

Transgenic constructs were prepared as described in Behura et al. [17]. In summary, FAIRE DNA elements of interest (Table 1) were PCR-amplified from *A. aegypti* genomic DNA and cloned into plasmid *pattBnucGFPs* (graciously provided by M. Halfon), a *phiC31*-enabled *Drosophila* transformation vector containing *EGFP* under the control of a minimal *hsp70* promoter. Transgenic *Drosophila* were produced at Rainbow Transgenic Flies, Inc. (Camarillo, CA) by injection into line *PBac{y[+]-attP-9A}VK00027* (Bloomington Stock Center #RRID:BDSC_9744 [19]). In each of two replicate experiments, tissue from 10 *w+* male and 10 *w+* female transgenic animals was collected and fixed as described previously [20]. In total, 80 antennae from each line were evaluated.

**Table 1:** FAIRE DNA elements assessed in *D. melanogaster* reporter assays

FAIRE DNA Element^a^	Flanks Gene no.^b^	Gene TSS
supercont1.174:341062-341799	*AAEL005776 (orco* ^c^ *)*	supercont1.174:357279
supercont1.1782:38974-39907	*AAEL016970 (Or1)*	supercont1.1782:39941
supercont1.671:130269-131236	*AAEL012254 (Or8)*	supercont1.671:131254
supercont1.199:700946-702158	*AAEL006301 (fru)*	supercont1.199:701824
supercont1.237:1279560-1280173	*AAEL007110 (Or16* ^c^ *)*	supercont1.237:1269860
supercont1.160:604315-605761	*AAEL005507 (acj6* ^c^ *)*	supercont1.160:435622
supercont1.123:863985-864746	*AAEL004572 (E93)*	supercont1.123:868,883
supercont1.54:975577-976601	*AAEL002359 (onecut)*	supercont1.54:924002

^a^The FAIRE DNA sequences shown [17] were assessed for their ability to drive EGFP expression in *D. melanogaster* reporter assays

^b^The flanking genes (gene number and name) and transcription start sites (TSSs) of the flanking genes are noted. Sequences correspond to *A. aegypti* scaffolds reference v.4, which was used in the FAIRE-seq investigation [17]. A subset of these elements (CREs associated with *orco*, *Or1*, *Or8* and *fru*) were used for subsequent generation of *A. aegypti* transgenic strains

^c^*Drosophila* reporter lines were initially described in Behura et al. [17] and are characterized in further detail in the present investigation

### *Aedes aegypti* transgenic construct generation

A derivative of the *PB-GAL4 ECFP* construct described in O’Brochta et al. [21] was generously provided by D. O’Brochta. The construct, which is compatible with both *pBac* and *phiC31* transgenesis, is marked with *ECFP* driven by a universal insect *3xP3* eye promoter [22]. This construct was modified through insertion of a new multiple cloning site (5'-CGC TAG CGC CGG CAG ATC TCC TAG G-3') between the *BgIII* and *NgoMIV* sites downstream of *pBacleft* and upstream of the open reading frame of *GAL4.* The addition of this multiple cloning site facilitated insertion of *fru*, *orco*, *Or1* or *Or8* FAIRE regulatory elements (Table 1), which were PCR-amplified, along with an upstream *hsp70* minimal promoter, from the *pattBnucGFP* constructs described above. Constructs were verified through restriction digestion and sequencing.

### *Aedes aegypti* transgenesis and transgenic line characterization

*Aedes aegypti* transgenic constructs described above were supplied to the Insect Transformation Facility (ITF) at the University of Maryland College Park facility, which generated the *A. aegypti* transgenics. The facility microinjected 70 *A. aegypti* (Liverpool strain) eggs (G0 animals) per construct. Following maturation, G0 adults were backcrossed to uninjected individuals. First generation (G1) offspring were reared and screened for ECFP expression. Transgenic G1 individuals were mated to generate transgenic strains. The resulting G2 individuals were screened *via* PCR with primers specific to the transgene for molecular confirmation of the transgenic line. G2 offspring were then crossed to establish a transgenic line, which was expanded and further characterized. Tissues were fixed and prepared as described [23]. *GAL4* expression driven by the FAIRE DNA regulatory element was monitored *via in situ* hybridization which was performed as described [24] and compared to endogenous gene expression patterns. Riboprobes for these studies were generated as described by Patel [25]; note that the *fru* riboprobe used in these studies corresponds to sequence that is conserved between the male and female splice forms [11]. For larval studies, in each of two replicate experiments, 40 antennae were dissected from 20 ECFP-positive larvae from each strain; in total 80 antennae were assessed per strain. For adult studies, 60 antennae from 30 ECFP-positive adults of each strain (15 males and 15 males) were assessed.

### Imaging and image processing

Following processing, tissues were mounted in glycerol and imaged on a Zeiss Axioimager equipped with a Spot Flex camera and Spot Digital Imaging software. Confocal imaging was performed at the IUSM Flow Cytometry and Imaging Core facility using a Zeiss 710 confocal microscope and Zen software. Images were analyzed with FIJI ImageJ and Adobe Photoshop CC 2014 software. Signal intensities between the *Or1-GAL4a versus b* and *Or8-GAL4a versus b* lines were evaluated through comparison of mean gray values (average signal intensity over the selected area) in each line for the specific cells noted in Fig. 2 (*n* = 25) and Fig. 3 (*n* = 15) using Fiji software. The cells selected for these analyses were chosen because they were easily recognized in the antennae of each mosquito assessed. Mean gray value data were statistically analyzed with a paired t-test.

## Results

### A screen for *A. aegypti* antennal CREs in *Drosophila*

**Fig. 1:**
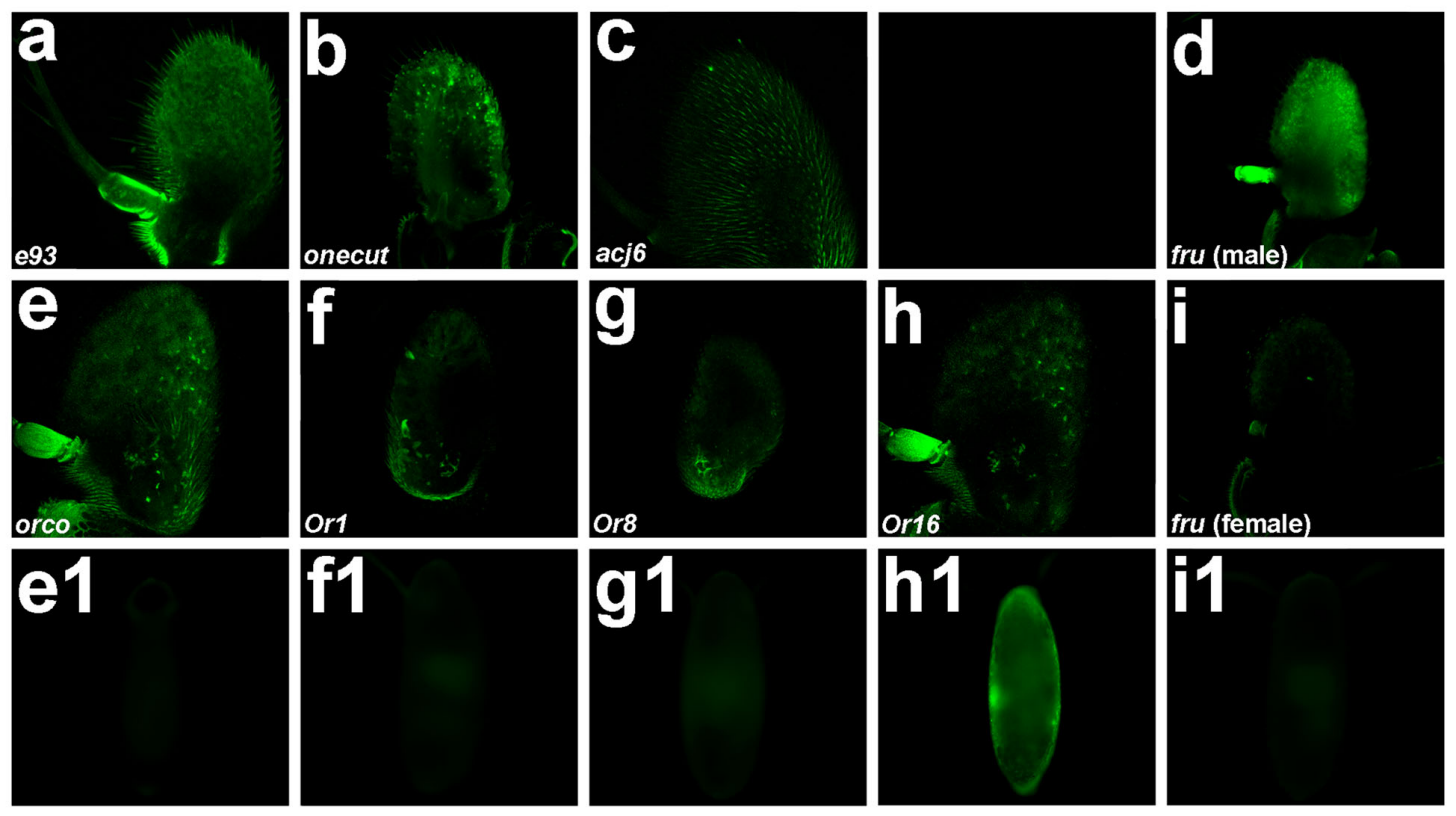
A *D. melanogaster* screen identifies *A. aegypti* CREs that promote gene expression in antennal ORNs. EGFP reporter expression driven by FAIRE DNA sequences flanking the indicated genes (*e93* in **a**, *onecut* in **b**, *acj6* in **c**, *fru* in **d**, **i**, and **i1**, *orco* in **e** and **e1**, *Or1* in **f** and **f1**, *Or8* in **g** and **g1**, *Or16* in **h** and **h1**) was assessed in a total of 40 female and 40 male adult antennae prepared from replicate experiments. EGFP expression patterns ranged from expression in all (**e**) or many (**a**- **c**) antennal ORNs to very specific subsets of ORNs (**f**-**h**). Sex-specific EGFP expression was detected in the adult male antenna (**d**; compare to female antenna in **i**). Embryonic expression of EGFP was detected in the *Or16* reporter line (**h1**), but not in the *orco* (**e1**), *Or1* (**f1**), *Or8* (**g1**) or *fru* (**i1**) *Drosophila* reporter lines. Proximal is oriented upward in **a-i**, and anterior is oriented upward in **e1**-**i1**

The *A. aegypti* FAIRE DNA data set [17] was assessed for regulatory elements residing adjacent to *A. aegypti odorant receptor* (*Or*) genes, as well as genes encoding transcription factors that function in the *A. aegypti* antenna to regulate *Or* gene expression [26]. It was hypothesized that FAIRE DNA elements flanking these genes, which are known to be expressed in *Aedes* antennal ORNs [26], would regulate gene expression in these neurons. In total, eight FAIRE DNA elements were identified and all were evaluated in this investigation (Table 1). The *A. aegypti* FAIRE DNA sequences were first cloned upstream of *enhanced green fluorescent protein* (*EGFP*) in a *Drosophila phiC31*-enabled transformation vector, and the resulting constructs were used for *Drosophila* transformation. All eight FAIRE DNA elements (Table 1) were confirmed to drive EGFP reporter expression in *D. melanogaster* (Fig. 1).

In an effort to select the most promising CREs for subsequent generation of *A. aegypti* transgenics (see below), the *Drosophila* reporter lines were characterized in detail. First, *D. melanogaster* reporters generated from FAIRE DNA residing upstream of the *A. aegypti E93*, *onecut* and abnormal chemosensory jump *(acj6)* genes (Table 1) were assessed. These genes encode transcription factors expressed in *A. aegypti* antennal ORNs, in which they are believed to play conserved roles in regulation of the *Or* expression code [26]. Antennal expression of EGFP was detected in all three of the *Drosophila* reporter lines (Fig. 1a-c). In the *E93 Drosophila* reporter (Fig. 1a), EGFP was expressed broadly in multiple adult ORNs. EGFP expression was restricted to a smaller subset of ORNs in the *onecut* reporter line (Fig. 1b). EGFP expression in the *acj6 Drosophila* reporter line, which had previously been documented in the *D. melanogaster* brain [17], was also detected in a subset of *D. melanogaster* adult antennal ORNs (Fig. 1c).

*Drosophila melanogaster* reporter lines generated using FAIRE DNA elements located adjacent to *A. aegypti Or* genes were also assessed (the *A. aegypti Or* genes were originally described in [27]). Two *Drosophila* reporter lines preliminarily characterized in the original study [17] had been generated with FAIRE DNA elements flanking the *A. aegypti orco* and *Or16* genes. The *orco* gene encodes the *A. aegypti* odorant coreceptor [28], while *Or16* encodes an odorant receptor protein of unknown function [27]. Behura et al. [17] had detected expression of GFP in the adult antennae of both reporter lines; both lines were characterized in further detail here. Analysis of the *orco* line confirmed that EGFP expression could be detected in all ORNs of the adult antenna (Fig. 1e). Further characterization of the *D. melanogaster Or16* reporter line confirmed EGFP expression in the fly antenna that was restricted to a subset of these adult ORNs (Fig. 1h)*.* Two additional *Drosophila* reporter lines, which contained FAIRE DNA upstream of the *A. aegypti Or1* (an odorant receptor of unknown function [27]) and *Or8* (which encodes a receptor for 1-octen-3-ol, an attractive odor emitted by hosts [29]) genes were generated and assessed. EGFP was also detected in the *D. melanogaster* adult antennal ORNs of these new reporter lines (Fig. 1f and g, respectively). EGFP expression was limited to subsets of ORNs in the *Or1* and *Or8 Drosophila* reporter antennae.

A FAIRE DNA element was also identified upstream of *A. aegypti fru* [17]. As discussed above, the *fru* gene, which is expressed in a sexually dimorphic pattern, is a key regulator of sex-specific behavior in *Drosophila* [30] and is believed to play comparable roles in *A. aegypti* [11]. Given the lack of tools available to study sexually dimorphic mosquito behaviors [2], this prospective CRE element was also selected for further analysis and used to construct a *D. melanogaster* EGFP reporter line. Reporter analyses demonstrated that although EGFP was expressed broadly in the adult male *D. melanogaster* antenna (Fig. 1d), it could not be detected in the female antenna (Fig. 1i). These findings suggested that this FAIRE DNA element isolated from *A. aegypti* might be a regulator of sex-specific antennal ORN gene expression.

### Generation of *A*. *aegypti* transgenics

The next task was to select high-priority CREs for analyses to be performed directly in *A. aegypti*. For these selections, it was determined that limited CRE activity, spatially and temporally, would be desirable. This could facilitate the specific manipulation of neurons of interest, for example through the expression of toxins for directed neural cell ablation, without concerns for unintended impacts on multiple tissues. Given the importance of sex-specific behaviors to mosquito biology and the transmission of disease-causing pathogens [2], the *fru* CRE, which did not drive reporter expression in *Drosophila* embryos (Fig. 1i1), was selected for further analysis in *Aedes*, in which it was hypothesized that it would promote post-embryonic sex-specific gene expression in the antenna. Regulatory elements upstream of *orco*, *Or1* and *Or8* drove specific EGFP expression in *Drosophila* reporter ORNs (Fig. 1e-g) and not in other reporter tissues (Fig. 1e1, f1, g1), and these elements were also selected for *Aedes* transformations*.* Based on the *Drosophila* reporter assays, it was hypothesized that these *A. aegypti* FAIRE elements would drive gene expression in all ORNs (*orco*), as well as subsets of neurons (*Or1* and *Or8*)*.* The selection of CREs that drive gene expression in both broad as well as narrow subsets of ORNs was intentional, as it is expected to promote flexibility in experimental design in *Aedes* in the future. The other elements listed in Table 1 were not prioritized for *Aedes* transgenic generation at this time, as it was predicted that these elements would be active in multiple tissues (Fig. 1h1) [17, 26].

**Fig. 2:**
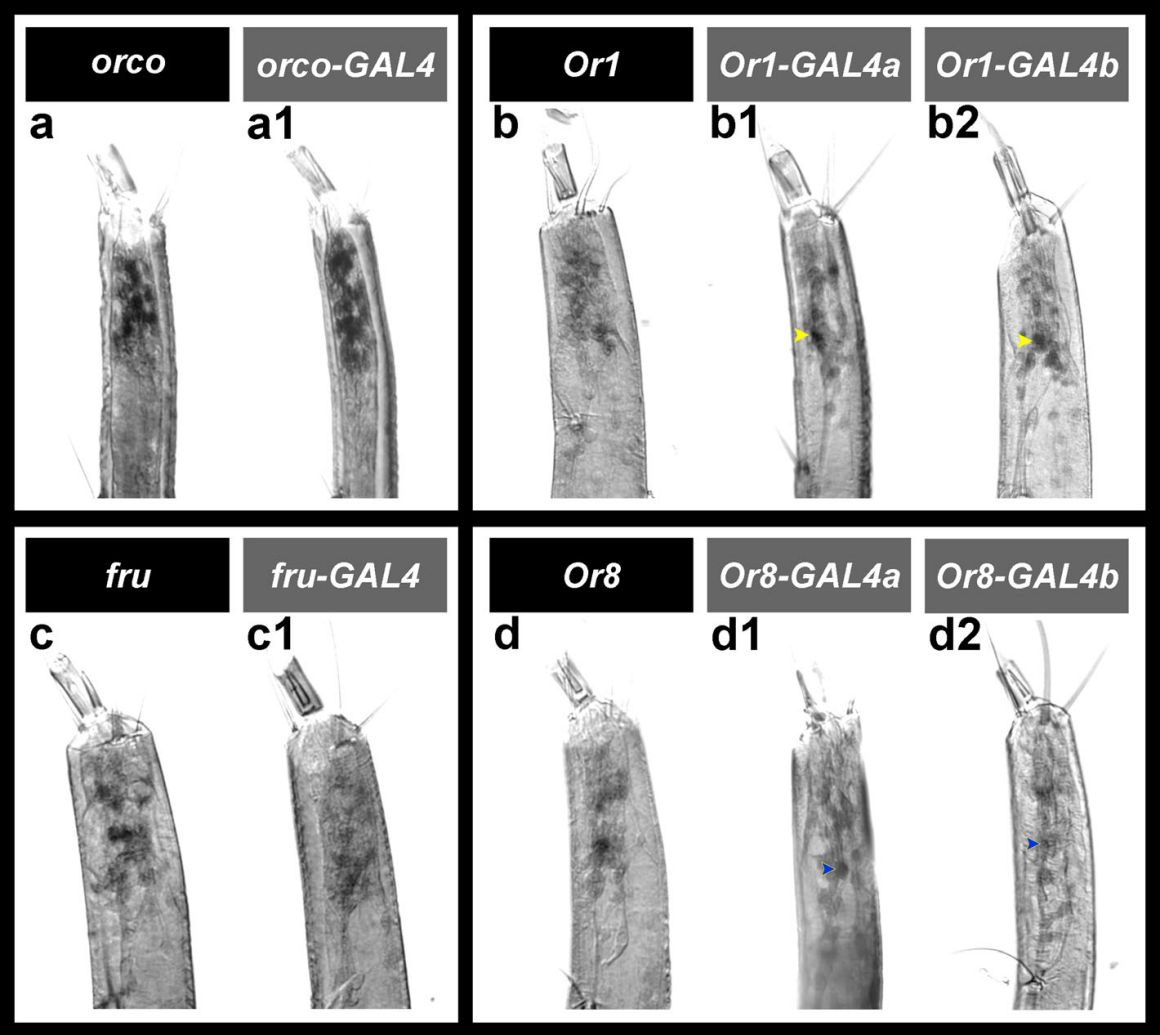
CRE activity in the *A. aegypti* larval antenna. FAIRE DNA elements associated with the *orco* (**a1**), *Or1* (**b1**, **b2**), *fru* (**c1**) and *Or8* (**d1**, **d2**) genes promote *GAL4* reporter expression in *A. aegypti* larval ORNs. The *orco* (**a1**) CRE promotes transgene expression in all larval ORNs, while the *Or1* (**b1**, **b2**), *fru* (**c1**) and *Or8* (**d1**, **d2**) CREs drive transgene expression in subsets of ORNs. These expression patterns are comparable to the patterns of native *orco* (**a**), *Or1* (**b**), *fru* (**c**) and *Or8* (**d**) transcripts in the larval antenna. CRE activity is comparable in two separate *Or1-GAL4* lines (*a* in panel **b1** and *b* in panel **b2**), as well as two separate *Or8-GAL4* strains (*a* in panel **d1** and *b* in panel **d2**). Mean gray value analyses for the cell marked by the yellow arrowheads in **b1** *versus* **b2** revealed no significant differences in transgene signal intensity levels (*P* > 0.05). Likewise, no differences in transgene signal intensity levels were detected for the cell marked by the blue arrowheads in **d1** *versus* **d2**. Proximal is oriented upward in all panels

**Fig. 3:**
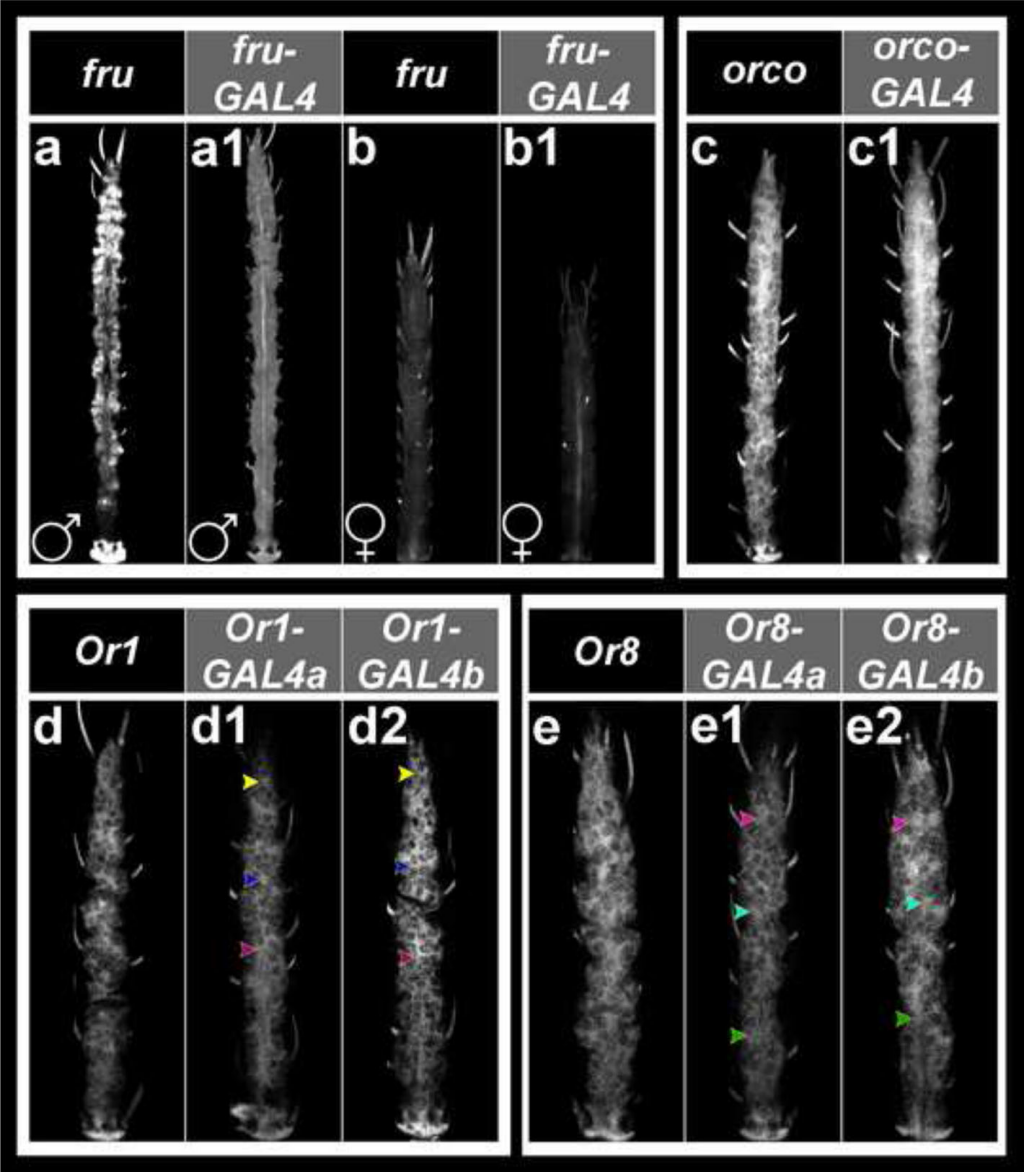
CRE activity in *A. aegypti* adult antennal ORNs. Expression of *GAL4* transcripts driven by FAIRE DNA elements adjacent to the *fru* (male in **a1**), *orco* (**c1**), *Or1* (**d1**, **d2**) and *Or8* (**e1**, **e2**) genes are comparable to expression of native *fru* (male in **a**), *orco* (**c**), *Or1* (**d**) and *Or8* (**e**) transcripts in the adult *A. aegypti* antenna. No *fru* transcript is detected in the *A. aegypti* female antenna (**b**), and *GAL4* expression is not driven by the *fru* CRE in the female antenna (**b1**). CRE activity is comparable in two separate *Or8-GAL4* lines (*a* in **e1** and *b* in **e2**), as well as two separate *Or1-GAL4* strains (*a* in panel **d1** and *b* in panel **d2**). Mean gray value analyses for the cells marked by the yellow, blue, or purple arrowheads in **d1** *versus* **d2** revealed no significant differences in transgene signal intensity levels (*P* > 0.05). Likewise, no significant differences were detected in the transgene signal intensity levels of the cells marked by the magenta, cyan, or green arrowheads in **e1** *versus* **e2** (*P* > 0.05). The *fru* (male in **a1**), *Or1* (**d1**, **d2**) and *Or8* (**e1** and **e2**) CREs are active in subsets of ORNs, while the *orco* (**c1**) CRE promotes gene expression in all ORNs. With the exception of **a** and **a1**, in which male antennae are shown, female antennae oriented proximal upward are displayed in all panels

A derivative of the *PB-GAL4 ECFP* construct described in O’Brochta et al. [21] was used for *piggyback* (*pBac*)*-*mediated *Aedes* transformation. The construct, which is marked by *ECFP* driven by a universal insect *3xP3* eye promoter [22], was modified through insertion of a multiple cloning site (MCS) upstream of the open reading frame of *GAL4,* which facilitated insertion of the selected FAIRE regulatory elements (Table 1), as well as an *hsp70* minimal promoter, upstream of *GAL4*. *GAL4* (like EGFP in the *Drosophila* assays) served as a reporter in this investigation. One *fru-GAL4* strain, an *orco-GAL4* strain, two *Or1-GAL4* lines and two *Or8-GAL4 A. aegypti* strains were successfully generated and characterized (Figs. 2 and 3).

### Characterization of *A. aegypti* transgenic strains confirms the discovery of regulatory elements that promote gene expression in *A. aegypti* antennal ORNs

The expression patterns of the *GAL4* transgene, which served as a reporter of CRE activity in this study, were assessed in the *A. aegypti orco-GAL4*, *Or1-GAL4* and *Or8-GAL4* strains. These were compared to the endogenous gene expression patterns of the *orco*, *Or1* and *Or8* receptors, which were first reported in larvae by Mysore et al. [26] (shown here for comparison in Fig. 2a, b, d) and were also characterized in the adult antenna (Fig. 3c-e) in the present investigation. In the *orco-GAL4* line, reporter expression, like endogenous *orco* expression in *A. aegypti* (Figs. 2a, 3c) and EGFP expression in the *D. melanogaster* reporter line (Fig. 1e), is detected in all *Aedes* antennal ORNs at both the larval (Fig. 2a1) and adult (Fig. 3c1) stages. Although the *orco* CRE did not promote EGFP expression outside of the *D. melanogaster* antenna, transgene expression was detected in the *A. aegypti orco-GAL4* brain (KM, unpublished observation).

Based on the *Drosophila* reporter assays (Fig. 1f, g), it was predicted that the *Or1* and *Or8* CREs would promote gene expression in subsets of ORNs. As predicted, the *Or1* CRE drove transgene expression in a subset of ORNs in the *A. aegypti* larval antenna (Fig. 2b1, b2) and the adult *A. aegypti* antenna (Fig. 3d1, d2) of the *Or1-GAL4a* and *b* strains. Comparable patterns of transgene expression were observed in the two strains (Fig. 2b1 *versus* b2; Fig. 3d1 *versus* d2), and transgene expression in the two *Aedes* strains matched that of endogenous *Or1* expression (larval expression is shown in Fig. 2b and adult expression in Fig. 3d). Furthermore, mean gray value comparisons of the cells marked in Fig. 2b1 *versus* b2 (t-test: *t*_(24)_ = 1.27, *P* = 0.22) or Fig. 3d1 *versus* d2 (t-test: *t*_(14)_ = 0.30, *P* = 0.77) detected no significant differences in transgene signal intensity levels between the *Or1-GAL4a* and *b* strains. Likewise, transgene expression patterns in the *Or8-GAL4a* and *b* lines were comparable (Fig. 2d1 *versus* d2 and Fig. 3e1 *versus* e2) and mimicked that of endogenous *Or8* gene expression (Fig. 2d; 3e) in both the larval (Fig. 2d1, d2) and adult (Fig. 3e1, e2) *A. aegypti* antenna. Levels of transgene expression were comparable between the *Or8-GAL4a* and *b* strains, with no significant differences detected in mean gray values for the cell marked in Fig. 2d1 *versus* d2 (t-test: *t*_(24)_ = 1.81, *P* = 0.08) or the cells marked in Fig. 3e1 *versus* e2 (t-test: *t*_(14)_ = 0.68, *P* = 0.51).

### Identification of a *fru* CRE that drives sex-specific gene expression in the *A. aegypti* antenna

As discussed above, a FAIRE DNA element associated with the *A. aegypti fru* gene drove male-specific reporter expression in a subset of *Drosophila* ORNs (Fig. 1d, i). This *A. aegypti* FAIRE DNA element was found to be active in a subset of larval (Fig. 2c1) and adult male (Fig. 3a1) ORNs of the *fru-GAL4 A. aegypti* strain. The pattern of transgene expression mimicked that of endogenous *fru* expression (Fig. 2c; 3a) in the *A. aegypti* larval (Fig. 2c1) and adult male (Fig. 3a1) antenna; however, transgene expression detected in the *fru-GAL4* adult male antenna was somewhat weaker than larval expression (Fig. 2c1) or that of endogenous *fru* expression (Fig. 3a). Neither endogenous *fru* expression (Fig. 3b) nor CRE activity (Fig. 3b1) were detected in the adult female antenna. Thus, an *A. aegypti* FAIRE DNA element associated with the *fru* gene promotes sex-specific gene expression in *A. aegypti* adult male antennal ORNs. It is possible that endogenous larval *fru* expression and CRE activity in the *fruGal4* strains was also sexually dimorphic in larval ORNs, but this was not assessed because *A. aegypti* males and females are not physically distinguishable as larvae.

## Discussion

### Identification of *A. aegypti* CREs that function similarly in the ORNs of divergently-related dipteran insects

The results of this investigation indicated that the *A. aegypti Or1, Or8*, *orco* and *fru* CREs identified through FAIRE-seq open chromatin profiling [17] can promote transgene expression patterns in the *A. aegypti* antenna that are comparable to the endogenous *Or1*, *Or8*, *orco* and *fru* gene transcript expression patterns in *A. aegypti* larval (Fig. 2) and adult (Fig. 3) antennae. These findings, in combination with our previous studies [17], provide evidence that FAIRE-seq is a powerful method for identification of CREs in mosquitoes. Given the success of the first study in *A. aegypti* [17], additional FAIRE-seq open chromatin profiling studies would be useful both in *A. aegypti* and other mosquitoes, as well as other non-model organisms. In an effort to identify CREs for multiple different tissue types, whole embryos were selected for our initial proof of concept studies in *A. aegypti* [17]. Analyses of these FAIRE-seq embryonic data (here and in [17]) confirmed that CREs for multiple different tissues were in fact identified, while also demonstrating that some of the CREs identified in embryos can function at later life stages. However, given that nucleosome positioning is influenced by multiple factors and can vary in different tissues and at different life stages (see reference [15] for a useful review), it will undoubtedly be useful to pursue tissue-specific FAIRE-seq open chromatin profiling directly in tissues of interest and at multiple mosquito life stages. For example, while the current investigation successfully identified multiple regulatory elements for the adult antennae, a FAIRE-seq investigation performed directly with tissue prepared from the adult antenna would likely uncover regulatory elements for additional *Or* genes. Likewise, although a sex-specific regulatory element was fortuitously identified in the present study, the direct comparison of open chromatin profiles in male *versus* female mosquito tissues of interest, including the antenna, would most likely identify additional CREs that promote sex-specific transcription and reveal insight into epigenetic mechanisms underlying sexually dimorphic traits.

As shown in this investigation, FAIRE-seq can be paired with secondary screening of elements of interest in *D. melanogaster* reporter assays to facilitate detection of CREs that promote gene expression in tissues of interest. The conservation of antennal CRE activity observed between *A. aegypti* and *D. melanogaster* in this investigation likely results from a conserved transcriptional regulatory code which is believed to regulate *Or* gene expression in the two species [26, 31]. In most cases, with *orco* being an exception, mosquito and fruit fly *Or* genes are not direct orthologues [27]. However, the functions of transcription factors that regulate *Or* gene expression in the antenna, including E93, Onecut, and Acj6, do appear to be conserved [26]. It is anticipated that since these *A. aegypti* CREs drive tissue-specific reporter expression in *Drosophila* that mimics the activity of the elements in *A. aegypti,* these CREs are likely to function similarly in other dipterans, including additional vector mosquito species that are more closely related to *A. aegypti* than are fruit flies. To test this, it may be interesting to transform other mosquitoes with the transgenic constructs generated in this investigation, which are compatible with *pBac* and *phiC31*-mediated transgenesis in multiple insect species. Finally, it is important to note that although the four antennal CREs characterized in this investigation appear to function comparably in *A. aegypti* and *D. melanogaster,* this will not be the case for all CREs. It is also important to uncover cases in which CRE function has diverged between the two species, and this will be a subject of future investigations.

Although *A. aegypti orco* is not expressed in the brain, ectopic CRE activity was detected in the *A. aegypti orco-GAL4* brain. Ectopic *GAL4* expression has been detected in the brains of other strains transformed with this construct ([21], KM and MDS unpublished) and is believed to be dependent on position-dependent variation in transgene insertion. Although the *PB-GAL4 ECFP* construct used in this investigation is compatible with both *phiC31* and *piggybac (pBac)* transgenesis [21], high-efficiency *phiC31* acceptor lines for site-specific integration are not openly available for *A. aegypti.* Use of site-specific integration would eliminate variation in transgene expression that can result from variable integration sites which can result when *pBac* transgenesis is utilized. Although several *Or1-GAL4*, *Or8-GAL4* and *fru-GAL4* strains which did not contain this ectopic brain expression domain were successfully generated in this investigation, it may nevertheless be useful to construct high-efficiency *A. aegypti attP* strains that would permit site-specific integration of constructs in the future.

### Developing a toolkit for manipulation of mosquito neurons

The *A. aegypti* strains generated in this investigation can significantly enhance neurogenetic analysis of olfactory system function in *A. aegypti.* In this study, CREs were cloned upstream of the *GAL4* transgene, which was used as a reporter for characterizing the activity of these CREs, but could also be used to drive expression of transgenes of interest in ORNs in the future. This will require generation of UAS responder lines for manipulation of neural gene expression and neural function in *A. aegypti*. It would be useful to generate UAS responder lines that express dsRNA to block gene function in specific neurons of interest. It would also be interesting to generate UAS responders that can function as neural tracers. For example, the plant lectin wheat germ agglutinin (WGA) has been used to trace neural circuits and mark synaptic activity in genetic model organisms, as it can be transported by axons and dendrites in both anterograde and retrograde directions, and it can be transferred across synapses [32]. *GAL4-*driven expression of *UAS-WGA* in the *Drosophila* nervous system, which can be detected through immunohistochemical staining with an antibody against WGA, has been used to uncover visual, motor neuron, and gustatory circuitries in *Drosophila* [32–34]. UAS constructs that drive targeted neural cell ablation would also be of interest. For example, an attenuated version of diptheria toxin is useful for ablation of specific cells [3] and has been used in neural cell death ablation studies [35]. Furthermore, expression of ion channels, toxins or genetically encoded proteins that activate or silence neural activity has revolutionized the study of neuroscience in genetic model organisms [3]. For example, optogenetics, light triggered neural activation, would be quite useful for analyses of mosquito neural function. Channel rhodopsins, light activated cation channels, the most widely used optogenetic tools for activation of neurons (reviewed by Venken et al. [3]), have yet to be reported in mosquitoes.

### Identification of a sex-specific regulatory element in *A. aegypti*

The FAIRE DNA element flanking the *A. aegypti fru* gene was found to drive sex-specific gene expression in the *A. aegypti* male antenna which mimicked the activity of this CRE in *D. melanogaster* reporter assays. These results suggest that some of the mechanisms which regulate sex-specific expression of *fru* are conserved between the two organisms. Recent studies [10] have demonstrated that the chromatin modulatory protein Alhambra restricts *fru* expression to specific neurons during development. Signaling from the ORs through CamK and a histone acetyl transferase protein then functions to maintain *fru* expression in specific ORNs [10]. It is possible that these mechanisms are conserved in *A. aegypti.* Moreover, in *D. melanogaster*, *fru* expression acts as a molecular marker to label neural circuits that regulate sex-specific behaviors [36]. If this is also the case in *A. aegypti*, the results of this investigation have provided both insight into the identity of at least some of the ORNs that are responsible for sexually dimorphic behaviors in *A. aegypti*, as well as a means of modulating gene expression in these neurons for the manipulation of sexually dimorphic behaviors.

In insects, olfaction is a critical component of sexually dimorphic behaviors. Fruit flies sense volatile pheromones and other odors that are critical for courtship behaviors *via* the olfactory system [37]. In *Drosophila,* the male splice-form of Fru, Fru^M^, is necessary and sufficient for regulation of sex-specific behaviors such as aggression and courtship in males [38]. *D. melanogaster* Fru is expressed in the neural circuit that promotes sexually dimorphic responses to the pheromone cis-vaccenyl acetate, a male-specific pheromone detected by OR67d-positive neurons that suppresses male-male and male-female courtship behavior [36]. Fru is also expressed in the Ir84a-positive class of neurons, which coordinate reproductive behaviors based on the availability of food resources [39]. A third Fru-positive class of ORNs expresses OR47b, which is responsible for detection of methyl laurate, a cuticular pheromone required for successful copulation [40–43]. Fru-positive neurons may behave in a similar fashion to regulate behavioral responses to pheromones and other odors that mediate sex-specific behaviors in *A. aegypti.* It will be interesting to assess which *Or* genes are expressed by Fru-positive neurons and to functionally characterize the role of Fru in *A. aegypti.* The *fru-GAL4* strain generated in this investigation could prove to be very useful in such investigations and may promote the elucidation of novel strategies for regulation of sexually dimorphic behaviors that contribute to mosquito reproduction or the spread of disease-causing pathogens.

## Conclusions

In conclusion, the results of this investigation demonstrate that FAIRE-seq, when paired with *D. melanogaster* reporter testing of FAIRE DNA elements of interest, is a high-yield method for identification of CREs that may function in mosquito tissues of interest. This methodology could be extended to other insect vectors of disease and other non-model organisms, in which discovery of CREs has been an ongoing challenge. The *A. aegypti* strains generated and characterized in this investigation expand the genetic toolkit available for the study of *Aedes* olfaction, including dissection of sensory contributions to sexually dimorphic mosquito behaviors that are critical for reproduction and the spread of disease-causing pathogens. The CREs identified in this investigation drive comparable olfactory neural expression patterns in both *A. aegypti* and *D. melanogaster* which likely result from a conserved transcriptional code that regulates *OR* expression in the two species [26, 31]. It is therefore likely that these CREs, which have been cloned upstream of *GAL4* in a transgenic construct that is compatible with transgenesis in multiple insect species, may function similarly in multiple dipteran insects, including other disease vector mosquito species.

## Abbreviations

*acj6*: *Abnormal chemosensory jump*
CRE: Cis-regulatory element
FAIRE: Formaldehyde-assisted isolation of regulatory elements
FAIRE-seq: Formaldehyde-assisted isolation of regulatory elements followed by sequencing
*fru*: Fruitless
G: Generation
*Or*: *Odorant receptor*
*Or1*: *Odorant receptor 1*
*Or8*: *Odorant receptor 8*
*orco*: *Odorant coreceptor*
ORNs: Olfactory receptor neurons
*pBac*: Piggybac
WGA: Wheat germ agglutinin
